# DDxNet: a deep learning model for automatic interpretation of electronic health records, electrocardiograms and electroencephalograms

**DOI:** 10.1038/s41598-020-73126-9

**Published:** 2020-10-02

**Authors:** Jayaraman J. Thiagarajan, Deepta Rajan, Sameeksha Katoch, Andreas Spanias

**Affiliations:** 1 Lawrence Livermore National Labs, Livermore, 94550 USA; 2 IBM Research AI, San Jose, 95035 USA; 3 SenSIP Center, Arizona State University, Tempe, 85281 USA

**Keywords:** Machine learning, Statistical methods

## Abstract

Effective patient care mandates rapid, yet accurate, diagnosis. With the abundance of non-invasive diagnostic measurements and electronic health records (EHR), manual interpretation for differential diagnosis has become time-consuming and challenging. This has led to wide-spread adoption of AI-powered tools, in pursuit of improving accuracy and efficiency of this process. While the unique challenges presented by each modality and clinical task demand customized tools, the cumbersome process of making problem-specific choices has triggered the critical need for a generic solution to enable rapid development of models in practice. In this spirit, we develop DDxNet, a deep architecture for time-varying clinical data, which we demonstrate to be well-suited for diagnostic tasks involving different modalities (ECG/EEG/EHR), required level of characterization (abnormality detection/phenotyping) and data fidelity (single-lead ECG/22-channel EEG). Using multiple benchmark problems, we show that DDxNet produces high-fidelity predictive models, and sometimes even provides significant performance gains over problem-specific solutions.

## Introduction

Precise differential diagnosis plays a crucial role in enabling robust decision-making and realizing effective patient care. In particular, interpreting time-varying recordings of electrical activity from the heart and brain, or collections of disparate measurements (i.e. electronic health records) is required frequently for clinical diagnosis and is central to understanding a gamut of abnormalities or detecting the onset of disease conditions. More importantly, a wide range of non-invasive diagnostic modalities, e.g. electrocardiogram (ECG) and electroencephalogram (EEG), have become highly prevalent because of their cost efficiency, thus leading to a deluge in diagnostic data being generated. Consequently, in the recent years, there has been a rapid growth in automation approaches for performing effective coarse/fine characterization of these measurements. Since it is almost impossible to recreate the complex decision-making process of clinical experts, when it comes to discriminating between multiple seemingly-similar conditions and handling inherent variations in the data, these automation methods rely almost entirely on data-driven pattern discovery^1^.

Though data-driven inferencing techniques have been broadly adopted across different streams of digital medicine, there is a strong emphasis for such tools in monitoring time-varying patient recordings, due to the cumbersome nature of manual interpretation. For example, processing short- and long-term EEG recordings is imperative for predicting the neurological state of a subject, such as detecting seizure events or early onset of abnormalities. Similarly, ECG interpretation is essential for detecting a variety of cardiac abnormalities, namely: atrial fibrillation (AF), myocardial infarction (MI) and arrhythmia^2^. In addition, retrospective analysis of health records enables phenotyping, where patient cohorts that belong to various disease conditions can be identified. Over the last decade, community-wide research efforts have led to the design of several predictive modeling solutions, in particular based on deep neural networks (DNNs), that can perform an accurate characterization, while being resilient to the inherent data challenges including sampling discrepancies, low-fidelity of measurements, class imbalances etc. A formal introduction to this large body of work can be found in broader survey articles such as^1,3,4^. These AI-powered solutions have produced unprecedented successes for a variety of challenging tasks in heart/brain health monitoring and phenotyping^2,5–12^. The research in this space has been accelerated by the curation of large-scale, open-source databases such as the TUH-corpus (Temple University)^13^, PhysioNet^14^ and Mimic^15^.

Though it might appear natural to directly adopt popular approaches from the computer vision/AI literature, e.g. recurrent models and convolutional neural networks (CNNs), to these problems, the unique challenges in dealing with clinical data has warranted the design of novel network architectures and improved training strategies. Consequently, state-of-the-art solutions often rely on a carefully chosen combination of classical feature-extraction techniques as well as modern representation learning paradigms^9,10,16–19^. A recurring design objective in these solutions is to build feature representations that are robust to inherent data variabilities and can effectively represent complex, multi-scale dependencies in the measurements^11,12^. Driven by the need for portable and rapid patient care, researchers have also explored the inclusion of additional constraints such as using only subset of the measurements, e.g. a single-lead ECG in lieu of 12 channels, to perform diagnosis, and found data-driven methods to be surprisingly effective^5^.

Despite these success stories, existing solutions for interpreting time-varying recordings are specialized to the task under consideration or the modality utilized, and rarely are they actually tested across different problems. Consequently, it is extremely challenging, even for machine learning (ML) practitioners, to choose an appropriate solution to deploy for a new problem at hand. In practice, the vast array of existing modeling choices makes this process highly cumbersome, and most often off-the-shelf solutions are found to be suboptimal. This motivates the design of a generic network architecture that can effectively operate across different scenarios, akin to a *multi-specialty* expert. Building such an architecture requires both the design of computing units that are suitable for different modalities and task characteristics, and rigorous empirical validation that it can perform competitively against specialized approaches.

### Proposed work

For the first time, we present a multi-specialty diagnosis model, DDxNet, that we demonstrate to be an effective architecture for a wide-range of diagnosis tasks, while enjoying the computational benefits of state-of-the-practice solutions. In the context of sequential data processing, the idea of dilated convolutional networks, which can automatically infer multi-scale features^20,21^ without the need for explicit feature pooling operations, provides a realistic opportunity for building an universal architecture for processing clinical time-series data. In this work, we find that *adaptively dilated causal convolutions* are an effective choice for the foundational computational unit to process clinical time-series data, and when coupled with dense architectures can provide significant modeling power. Our empirical studies clearly evidence that our approach, with no additional architecture tuning, outperforms state-of-the-art solutions specifically designed for different benchmark tasks. In addition to producing highly effective predictive models, DDxNet enables rapid prototyping of solutions in practice.

**Figure 1:**
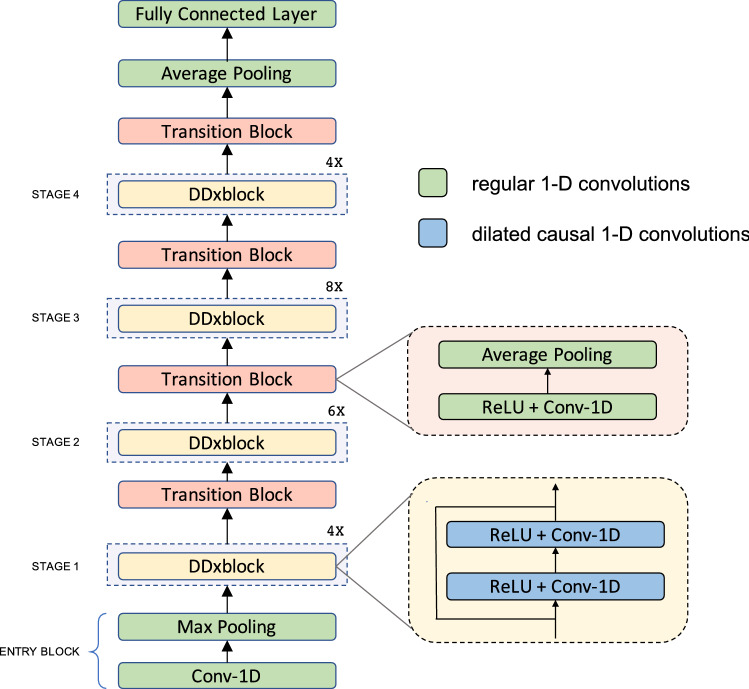
An illustration of the proposed DDxNet architecture. DDxNet builds a densely connected network with dilated causal convolutions, wherein the dilation factor is adaptively adjusted for extracting multi-scale features. Each DDxblock is comprised of a bottleneck layer and a convolutional layer designed according to the *growth rate* hyper-parameter. Each processing stage is followed by a transition block which performs temporal aggregation prior to invoking the next stage.

In order to realize a generic architecture for different benchmark problems, DDxNet leverages the following key algorithmic contributions: CNNs comprised of causal convolutions coupled with dense connections, adaptive dilation to incorporate multi-scale feature extraction, and carefully chosen regularization and optimization recommendations. The model architecture, as illustrated in Fig. 1, is obtained by stacking three different types of fully convolutional sub-networks—(i) *entry* block for initial processing of multi-variate measurements; (ii) DDxblock block with adaptive dilation, i.e. increasingly expanding receptive fields, to enable principled learning of multi-scale features and to support feature reuse during model optimization; and (iii) *transition* block for performing feature aggregation and dimension reduction. Note that, the use of causal convolutions in all parts of the network enables improved modeling of non-stationary data. The specifics of the architecture and recommended training strategies are elaborated in the “Methods” section.

### Results

We evaluated DDxNet using a disparate set of challenging diagnosis tasks, namely (i) abnormality detection (e.g. seizure) using EEG^13^, (ii) myocardial infarction diagnosis from ECG^22^, (iii) arrhythmia classification using single-lead ECG^23^ and (iv) acute care phenotyping using EHR^24^. These benchmark tasks encompass a wide-range of real-world challenges including varying data sizes, different task complexity, and discrepancies in measurement fidelity (single-lead ECG to 22-channel EEG recordings). To further emphasize the generic nature of DDxNet, we used the same architecture and training hyper-parameters across the different use-cases.

DDxNet produces state-of-the-art performance on the challenging abnormality detection task with EEG signals by surpassing the best reported recall rates by a significant margin (4%). Similarly, in myocardial infarction diagnosis using single-lead ECG, DDxNet achieves dramatic improvements by producing an accuracy of 99.7% on the PhysioNet PTB dataset. In the challenging multi-class arrhythmia classification task, our approach improves over the best-known results in the literature, even while using a subset of measurements (only 2 ECG leads). Finally, for phenotyping with EHR data of ICU patients^15^, DDxNet is highly competitive with approaches based on RNNs^25^ and attention models^26^. Interestingly, DDxNet is consistently superior over off-the-shelf alternatives for generic architectures, such as residue networks^9^ and temporal convolutional networks (TCN)^21^, that are commonly utilized in computer vision and language processing applications. Our empirical studies clearly evidence the general applicability of DDxNet as an architecture for practical diagnostic tasks. This finding provides tremendous opportunities to significantly reduce time or resources spent in engineering network architectures and to shift the focus to better understanding the gaps between AI-driven diagnosis and decision process of experts, which is critical for deploying clinical models in the real-world.

## Benchmark problems and data

**Figure 2:**
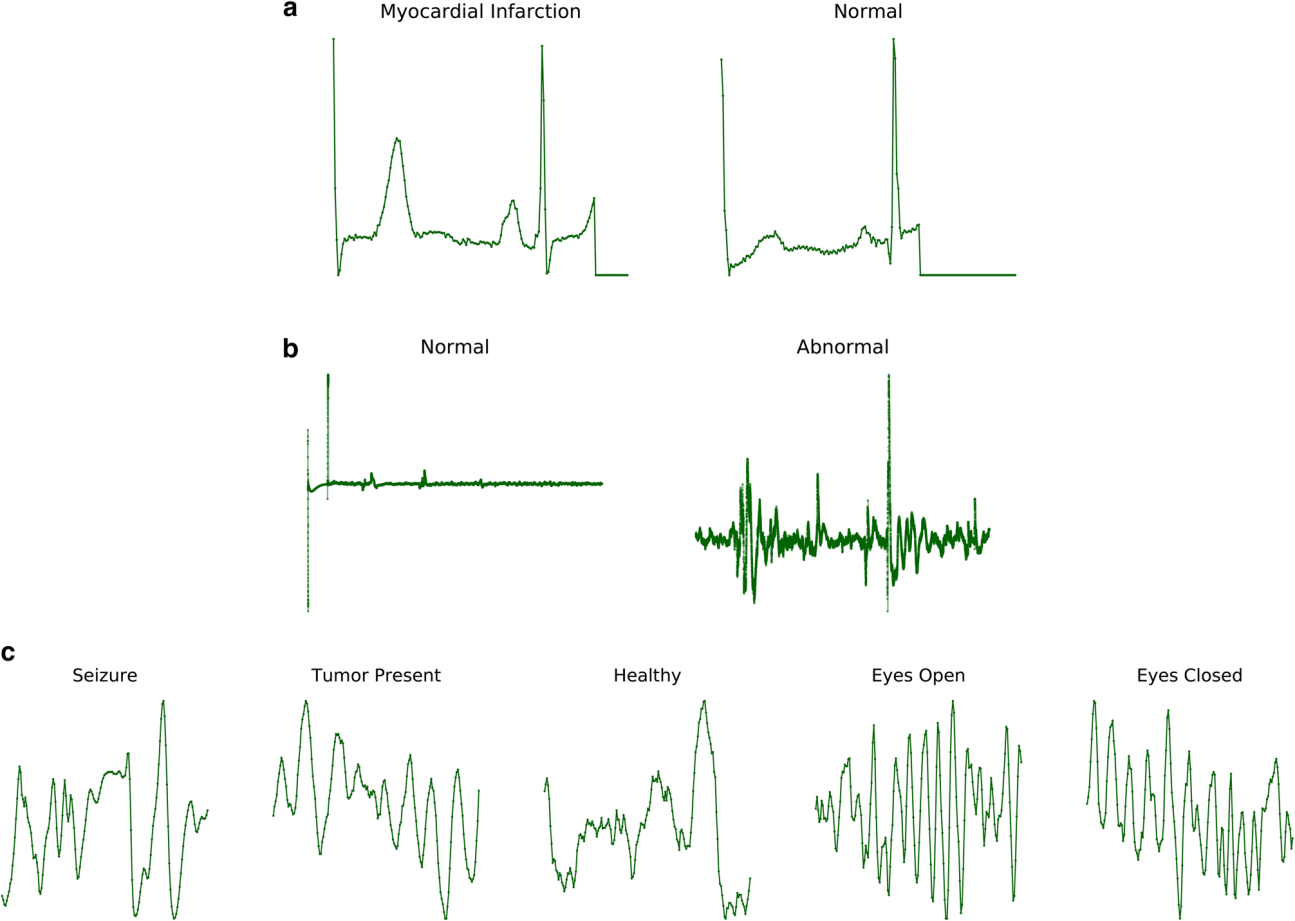
Example data from the different benchmark diagnosis problems considered in this paper: (**a**) Myocardial Infarction detection; (**b**) Arrhythmia classification; (**c**) EEG abnormality detection. Note that, for the case of *EEG Abnormality Detection*, we show only one of the 22 channels.

In pursuit of designing a generic predictive modeling architecture for different clinical diagnosis tasks, we consider a set of benchmark problems, which vary in the data modality utilized, the required degree of characterization, and the assumptions on data fidelity. These benchmarks broadly represent challenges commonly encountered in cardiovascular/neurological diagnosis and phenotyping, and are typically solved using highly specialized ML solutions. With DDxNet, our goal is to provide an all-encompassing approach that can seamlessly transition across different scenarios, and to quantitatively evaluate its effectiveness against strong problem-specific baselines from the literature. Figure 2 illustrates examples from each of the benchmark datasets.

### EEG-based abnormality detection

The complex dynamics of a brain system can be viewed through EEG signals recorded by non-invasively placing electrodes on the scalp. Clinical studies focus on determining correlations between these signals and observable action during abnormal responses. Typically, an EEG technician reviews numerous montages looking for discrepancies in various frequency bands, starting with the alpha (7.5–12 Hz) component responsible for relaxation. The multi-dimensional nature of EEG signals carry critical frequency information, but similar content could mean different outcomes depending on which locations in the brain they are being picked up from. Consequently, feature representations that preserve both frequency and spatial characteristics have proven to be successful in prediction tasks. Further, lengthy recordings tend to be highly noisy due to active patient movements such as eye blinking. Finally, the inherent variabilities in measurements based on patient demographics such as age and gender play a significant role in the overall interpretation of EEG patterns. For example, an infant showing flat lines and other turbulent regions in the recordings is normal, while interpreting the same patterns if it came from an older adult could mean abnormal. A typical process of EEG interpretation involves analyzing potential epileptic activity, spikes, sharp waves, background slowing, asymmetrical behavior and natural occurrences of awake, sleep and drowsy states. This process is known to be cumbersome, given the volume and long durations of recordings, as subjects are monitored for 48 to 72 h on average. Consequently, a first step in automating EEG screening is to accurately detect abnormalities.

#### Data and pre-processing

Building effective abnormality detection approaches has remained a challenge due to the lack of well-annotated datasets, owing to the labor-intensive annotation process. Recently, tremendous effort has been made in curating an open-source dataset, the TUH abnormal corpus, to enable machine learning research in EEG interpretation^13^. This dataset is comprised of EEG sessions from 2993 patients, out of which 1521 are normal and 1472 are abnormal. Each EEG record includes 22 channels, sampled at 250 Hz in an average reference (AR) configuration spanning at least 15 min long. It is widely accepted in the community that earlier segments of an EEG session are less likely to be corrupted by changes in recording conditions, when compared to the later stages^27^. Consequently, in our setup, each sample in our data is constructed as the first 5 min snapshot of a subject’s EEG. Subsequently, we convert the snapshot into a transverse central parietal (TCP) montage configuration, which is a common protocol for abnormality detection. The pre-processing step involves computing Mel Frequency Cepstral Coefficients (MFCC)^28^ for each channel, a popular feature extraction technique used in time-varying signal processing—we first resample the data from 250 to 100 Hz, and use a 2000-point FFT with 40 filters and an overlap of 100 timesteps, thus resulting in a sample of 880 channels and 301 timesteps.

### ECG-based arrhythmia classification

A crucial step towards monitoring cardiovascular health is to detect the presence or absence of abnormal rhythms in ECG. This typically involves delineation of different wave segments (P-wave, QRS-complex, T-wave) and their relationships from a standard 12-channel ECG recording. Several cardiac diseases including atrial fibrillation, ventricular flutter, tachycardias and left/right bundle branch blocks (BBB) manifest as anomalous deviations in ECG channel configurations, where each lead provides a unique perspective on the electrical activity of the heart. For example, leads II, III, and aVF are used to detect inferior myocardial infarction, while leads V1 and V6 are used for bundle branch block^8^. Despite that, in telemetry and other ambulatory settings only a subset of channels are accessible, making the task of abnormality detection more challenging^9^. Consequently, the problem of arrhythmia detection is often formulated as classifying heartbeats, using only single-channel ECG, into one of the 5 categories prescribed in the association for advancement of medical instrumentation (AAMI) EC57 standard. The list of abnormalities are {N, S, V, F, Q} that can be broadly mapped to {BBB, atrial pre-mature, ventricular premature, fusion beats, paced beats} respectively.

#### Data and pre-processing

The MITBIH database^23^ contains 47 subjects, both inpatients and outpatients from Boston’s Beth Israel Hospital. Each recording is a two-channel ECG, 48 h long and sampled at 360 Hz. However, for the task of arrhythmia classification, the ECG data is initially pre-processed by creating a representation for every heartbeat using the following protocol^10^: the lead II signal is resampled to 125 Hz and split into normalized windows of 10 s. Subsequently, for each snapshot, we detect the R-peaks and R-R intervals, essentially generating a heartbeat signal, which is finally classified into one of the five categories described earlier.

### ECG-based myocardial infarction detection

Detecting myocardial infarction (MI) is one of the most crucial problems within the computerized ECG interpretation community. There have been several solutions proposed to solve this task using both 12 ECG channels as in^29,30^ and^19^, as well as a limited subset of channels as shown in^8,18^. The standard 12-lead ECG depicts evidence of ischemic heart diseases that predominantly occur due to the narrowing of blood vessels caused by atherosclerosis. Abnormalities in ECG segments such as the T-wave and Q-wave in addition to ST-elevation are typical signs of myocardium damage leading to a myocardial infarction (MI), commonly referred as a heart attack. Infarction could occur in different regions of the heart, and are picked up by the corresponding ECG leads^9^. In this problem, we consider the challenging task of detecting all variants of MI (including lateral, septal and posterior MI) using only a single channel of ECG (lead II).

#### Data and pre-processing

The Physionet PTB database^22^ includes 148 subjects with MI and 52 subjects with normal heart rhythms. Each ECG record is 30 s long, sampled at 1 KHz. Similar to the case of arrhythmia classification, we use a single channel ECG (lead II) resampled to 125 Hz.

### EHR-based phenotyping of ICU patients

The process of acute care phenotyping involves predicting likely disease conditions based on a patient’s ICU measurements, thereby enabling a wide-range of capabilities including comorbidity detection, diagnosis and risk assessment. Typically, phenotypes are identified by clinical experts and defined using the ICD-9 billing codes. In this study, we retrospectively classify phenotypes as the billing codes do not have timestamps. In other words, the exact occurrence of symptoms and diseases for each patient is unknown. We consider a total of 25 disease conditions, including 12 critical ones such as respiratory failure or sepsis, 8 chronic conditions such as diabetes and atherosclerosis, and 5 ‘mixed’ conditions such as liver infections. It is important to note that a patient can be associated with multiple conditions, and hence phenotyping is posed as multi-label classification. While this MIMIC-III benchmark contains only numerical values from test measurements, DDxNet can be applied even to EHR data with diverse entities including symptoms and drugs. In such scenarios, following common practice^31^, we will first map clinical concepts (e.g. symptoms) into vector representations (concept embeddings) and then subsequently build a predictive model using DDxNet.

#### Data and pre-processing

The MIMIC-III benchmark dataset^15^ contains a cohort of 33,798 unique patients with a total of 42,276 hospital admissions and ICU stays. Each patient’s EHR is transformed into a 76-dimensional time-series (corresponding to the total number of measurements) that encompasses ICU measurements including diagnostic codes, blood pressure, heart rate, Glasgow coma scale (GCS) etc. In our setup, each sequence is comprised of 256 time steps, where the measurements were acquired every 2 h.

## Results

Through rigorous empirical analysis with the benchmark problems, we find that, with the same underlying network architecture, DDxNet produces high detection rates, often outperforming even problem-specific state-of-the-art solutions, thus motivating its adoption as a generic approach for clinical diagnosis from time-varying measurements. Note that, all results reported in this paper were obtained using the standard train-test data splits prescribed in each of the benchmark datasets.

Typically, in EEG-based abnormality detection systems, a high-quality solution is characterized by a high recall of the abnormal cases, while producing a satisfactory overall accuracy. Hence, we utilize accuracy, recall and precision for the abnormal cases as the evaluation metrics.

$$\begin{aligned} \text {Acc}=\frac{tp + tn}{tp + fp + fn + tn}; \quad \text {Rec} = \frac{tp}{tp + fn}; \quad \text {Prec} = \frac{tp}{tp + fp}, \end{aligned}$$

where *tp*, *fp*, *fn*, *tn* correspond to the number of true positives, false positives, false negatives and true negatives respectively. Table 1 reports the abnormality detection performance on the challenging *TUH* corpus obtained using DDxNet in comparison to state-of-the-art baselines. Most importantly, DDxNet provides the highest recall so far on this dataset, with an improvement of 4% over the current state-of-the-art *ChronoNet*, without compromising on the overall accuracy. Note that, this performance improvement can be attributed to the key architectural innovations in our approach. Although approaches such as temporal convolution networks (TCN)^21^ and the ResNet^5,32^ are expected to be effective in clinical diagnosis tasks as a general solution, DDxNet outperforms the former by $\sim \,9.5$% improvement in recall, while significantly improving over the latter (13.5% improvement in recall).

**Table 1:** *EEG Abnormality Detection*—performance of DDxNet on the publicly available TUH data corpus.

Method	Acc (%)	Rec (%)	Prec (%)
Lopez et al.^27^	78.8	75.4	77.8
Andreotti et al.^32^	82.2	73.8	85.32
Bai et al.^21^	82.2	77.8	82.35
Roy et al.^11^	**86.6**	83.46	**86.7**
DDxNet	**86.6**	**87.3**	84.0

For comparison, we report the results obtained using several state-of-the-art baselines. The best numbers are shown in bold.

In the arrhythmia classification task, ECG signal abnormalities are assigned to one of the 5 types of abnormalities elaborated in the setup. DDxNet was evaluated and compared to several competitive baseline models that have used both traditional domain expert-based feature engineering as well as convolutional and recurrent style neural networks. To obtain a holistic evaluation of the prediction quality, we consider the overall accuracy and f1-score metrics. Note, the f1-score can be measured as

$$\begin{aligned} \text {f1}=2\cdot \frac{\text {Prec}\cdot \text {Rec}}{\text {Prec} + \text {Rec}}. \end{aligned}$$

**Figure 3:**
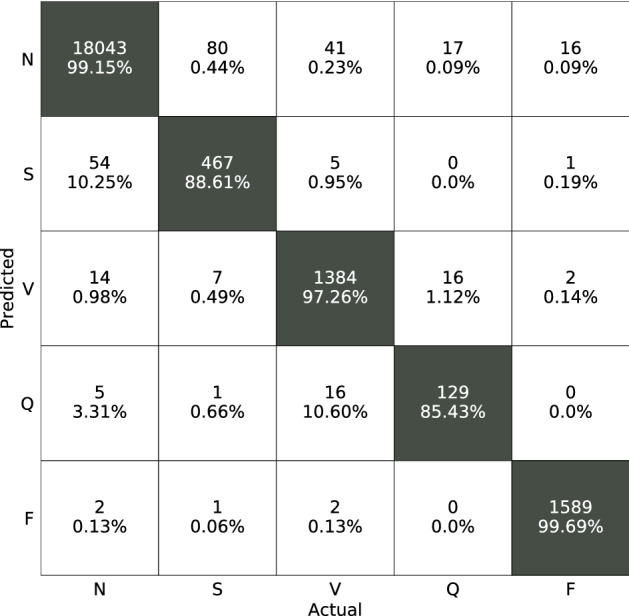
Confusion matrix obtained using DDxNet for the 5-category abnormality detection task (*Arrhythmia classification*).

Despite an appreciable imbalance in the label distribution of the MIT-BIH dataset, the performance of DDxNet exceeds that of existing approaches, achieving an improvement of 5% in terms of prediction accuracy over the state-of-the-art baseline^10^. The striking observation is that, the performance reported were obtained using a single channel ECG (lead II), which clearly emphasizes the effectiveness of DDxNet, even when the data fidelity is low. Further, as showed in Table 2, DDxNet leads to major improvements over existing convolutional neural network solutions^10,18^. The confusion matrix from our approach, showed in Fig. 3, evidences the ability of DDxNet in handling severe class imbalances.

**Table 2:** *Arrhythmia Classification*—performance of DDxNet with single-lead ECG.

Method	Acc (%)	f1-score
Kachuee et al.^10^	93.4	–
Bai et al.^21^	97.7	0.864
Acharya et al.^18^	93.5	–
Liu et al.^37^	94.4	–
DDxNet	**98.5**	**0.927**

Best numbers are shown in bold.

Given the amount of variabilities within the manifestations of myocardial infarction, using the entire 12 channel ECG has been long considered to be essential. However, using the pre-processing described earlier that involves simple heartbeat signal extraction, DDxNet accomplishes near-perfect detection using just a single channel ECG (lead II) as shown in Table 3, outperforming all other competing solutions including those that use all 12 channels^29,30^. Note, similar to the EEG-based abnormality detection experiment, we use the accuracy, precision and recall metrics.

**Table 3:** *Myocardial Infarction Detection*—performance of DDxNet in detecting Myocardial Infarction from ECG recordings.

Method	Acc (%)	Rec (%)	Prec (%)
Kachuee et al.^10^	95.9	95.1	95.2
Acharyaet al.^18^	93.5	93.7	92.8
Kojuri et al.^29^	95.6	93.3	97.9
Sharma et al.^30^	96	93	99
Bai et al.^21^	98	98.7	98.6
Nils et al.^19^	–	93.3	93.6
Rajan et al.^8^	86	96	–
Reasat et al.^38^	84.54	85.33	–
DDxNet	**99.7**	**99.7**	**99.9**

Interestingly, even with a single lead ECG, DDxNet achieves near-perfect detection.

For the task of phenotyping using EHR, making accurate disease predictions usually requires modeling longer term dependencies. Moreover, the disproportionate class labels cause overfitting of models to the training sets. Following standard practice^15,25^, we evaluate using the three following metrics based on the Area Under ROC Curve (AUROC): (i) micro-averaged AUROC that estimates an overall averaged score across all classes; (ii) macro-averaged AUROC that produces class-specific estimates; and (iii) weighted-average AUROC that considers disease prevalences while aggregating across classes. In general, AUROC measures the area under the ROC curve created by plotting true positive rates (tpr) against false positive rates (fpr), defined as follows:

$$\begin{aligned} \text {tpr} = \frac{tp}{tp + fn}; \quad \text {fpr} = \frac{fp}{fp + tn}. \end{aligned}$$

Table 4 evidences that DDxNet produces state-of-the-art performance when compared against baseline methods based on recurrent neural networks^15^ and attention models^26^, specifically designed for this task. Furthermore, we observed that the performance of DDxNet was consistent for varying lengths of the observation window (72, 96, 128 and 256 time steps), further emphasizing its effectiveness as a practical solution.

**Table 4:** *Phenotyping using health records*—performance of DDxNet in cohort discovery from EHR for different conditions.

Method	Micro AUROC	Macro AUROC	Weighted AUROC
Logistic Regression^15^	0.80	0.74	0.73
Song et al.^26^	0.81	0.76	0.75
LSTM^15^	**0.82**	**0.77**	**0.76**
C-LSTM^15^	**0.82**	**0.77**	–
DDxNet	**0.82**	**0.77**	**0.76**

## Discussion

The ability of data-driven models to deal with large-scale, complex clinical data could lead to unprecedented breakthroughs towards improving patient care. However, for this potential to materialize into new insights, ML models must be consistently effective for different diagnosis tasks, and must handle challenges that arise in real-world clinical settings. While the majority of the community’s focus has been on curating annotated datasets that represent these challenges, designing predictive modeling solutions that are broadly applicable to different scenarios and varying data modalities remains to be addressed. Given the rapid developments in the fields of AI and deep learning, it is becoming increasingly difficult, and often time-consuming, for practitioners to build customized solutions. Though there exists several architectural choices for every sub-problem under the broad umbrella of AI-driven diagnosis, rarely are their generalization capabilities to unseen data and vulnerabilities to population shifts well-understood. As the complexity of ML techniques increases with new algorithmic innovations, the inability to obtain a holistic understanding of strengths and weaknesses of models makes it challenging, and sometimes counter-productive, to deploy such models in the real-world.

This indicates a crucial gap in the current practice of AI for healthcare, wherein there needs to be a paradigm shift from fine-tuning model architectures for the limited labeled data at hand to better understand a model’s behavior in scenarios different from their training regime and to verify that predictions arise from generalizable patterns rather than artifacts or biases in the training data. To the best of our knowledge, this is the first work aimed at designing a generic model architecture that is suitable for a broad range of diagnosis tasks with time-varying clinical data, thus eliminating the need for scenario-specific architecture design. In particular, we presented DDxNet, a foundational architecture that we demonstrated to be effective across challenging benchmark problems, spanning different data modalities, data fidelities and diagnosis tasks.

In summary, we believe that generic solutions, such as DDxNet, that are broadly applicable to different problems of interest will foster increased emphasis on other crucial research avenues in AI for healthcare such as incorporating uncertainty quantification, understanding model behavior under domain shifts, enabling model interpretability, ensuring fairness etc. This is imperative to quantify the gap between AI-driven diagnosis and decision process of clinical experts, and thus build more meaningful healthcare models.

## Methods

DDxNet is a fully convolutional architecture, similar to several existing state-of-the-art^3,5,9^ solutions in clinical diagnosis, which are known to be superior to conventional methods relying on hand-engineered features. While resnet-style solutions have produced unprecedented success with challenging problems in clinical diagnosis, e.g. atrial fibrillation detection from ECG and seizure onset prediction using EEG measurements, DDxNet utilizes dense connections between stacked convolutional layers^33^, along with multi-scale feature extraction, for improved modeling. While a systematic control of how the network expands provides meaningful abstractions of patterns in time-varying data, including dense connections ensures maximal information flow between layers in the network. In contrast to resnet-style architectures^5^, which combine features through summation before they are forwarded to the next layer, we combine features by concatenating them. Through this connectivity pattern, the multi-scale abstraction can be entirely accessed by the later layers, thus leading to much improved feature representations. Further, as argued in^33^, using dense connections will ensure that the features are reused, thus avoiding redundancy in the learned feature maps.

### Architecture

We now describe the architectural choices required to design DDxNet, illustrated in Fig. 1DDxNet is built by stacking blocks of convolutional layers designed to capture multi-scale patterns in temporal data through dilated causal convolutions, and to compound meaningful abstractions from those patterns.

#### A multi-stage fully convolutional model

The architecture is comprised of a sequence of repeating convolutional blocks—the outputs of a block are concatenated with the input to that block, before being forwarded to the next convolutional block. DDxNet is based entirely on $1-D$ convolutions that computes filter activations only in the temporal dimension. Referred to as a DDxblock, each block contains a bottleneck convolution layer with kernel size $k = 3$, followed by another convolution layer. Each DDxblock block produces the same number of output features, and the *growth rate* parameter controls the rate at which the network expands. Our network contains a total of 4 processing stages, wherein each of the stages contains 2, 6, 8 and 4 DDxblocks respectively. Prior to invoking the multiple stages of densely connected convolution layers, the input is processed using a convolutional layer with $k=7$ and a max pooling layer.

Note that, after each stage, we include a *transition block*, that performs temporal aggregation through average pooling with stride $s = 2$, and then carries out channel-wise dimensionality reduction using a bottleneck convolution layer with $k = 1$. The resulting features from the transition block in the final stage are processed through an average pooling layer to produce a single feature vector for the entire sequence. The final classification layer is implemented using a single fully connected layer with *softmax* activation. In contrast to CNN architectures used in vision applications, we do not perform batch normalization in any stage of the network. We found in our empirical studies that using batch normalization resulted in a poorer convergence behavior during training.

#### Adaptive dilation

Dilated convolutions have become an integral part of several successful sequence-data processing approaches—examples include *Wavenet*^34^, segmentation networks^20^ and temporal convolutional networks^21^. Basically, dilated convolutions are convolutions with expanded receptive fields. As a result, stacking dilated convolutions with increasing dilation factors amounts to obtaining a multi-scale abstraction of the input signal. In a standard 1-D convolution with $k = 3$, the feature activations are obtained based on the adjacent time-steps. When the dilation factor is set at *d*, a dilated convolution with the same kernel size applies the filter to every $(d-1)$th instead of the directly adjacent time-steps. This effectively captures a larger context without the need to explicitly perform down-sampling of the signal. Every DDxblock is implemented using dilated convolutions, wherein the dilation factor is increased exponentially within each stage as follows:

1 $$\begin{aligned} d_i = \min (128, 2^{i+2}), \end{aligned}$$

where *i* denotes the index of the DDxblock in each stage. This enables a principled way to extract multi-scale features from the time-varying clinical data and effectively back-propagate gradients through the multiple stages. Note that, we set the maximum dilation factor 128, since for the most commonly adopted sampling rates in clinical recordings, larger contexts produce features that do not generalize well. This observation corroborates well with the results in a recent work^26^, where the authors designed an attention-only architecture for clinical modeling, and found that larger contexts while computing the attention weights led to poorer generalization.

#### Causal convolutions

Another important feature of DDxNet is that it employs causal convolutions. With causal convolutions, the activations that a layer produces at time-step *t* of the signal depends only on data obtained before *t*. While this is commonly used in speech synthesis models such as the Wavenet^34^, their importance in diagnostic models has not been well-studied yet. In particular, when there are repetitive patterns in the measurements, enforcing causality while computing the receptive field will not lead to improved representations. However, when the data under consideration is highly non-stationary, enforcing causality can be highly beneficial. Hence, DDxNet employs causal convolutions in all stages, while retaining normal convolutions (without causality or dilation) in the entry and transition blocks.

#### Training

**Figure 4:**
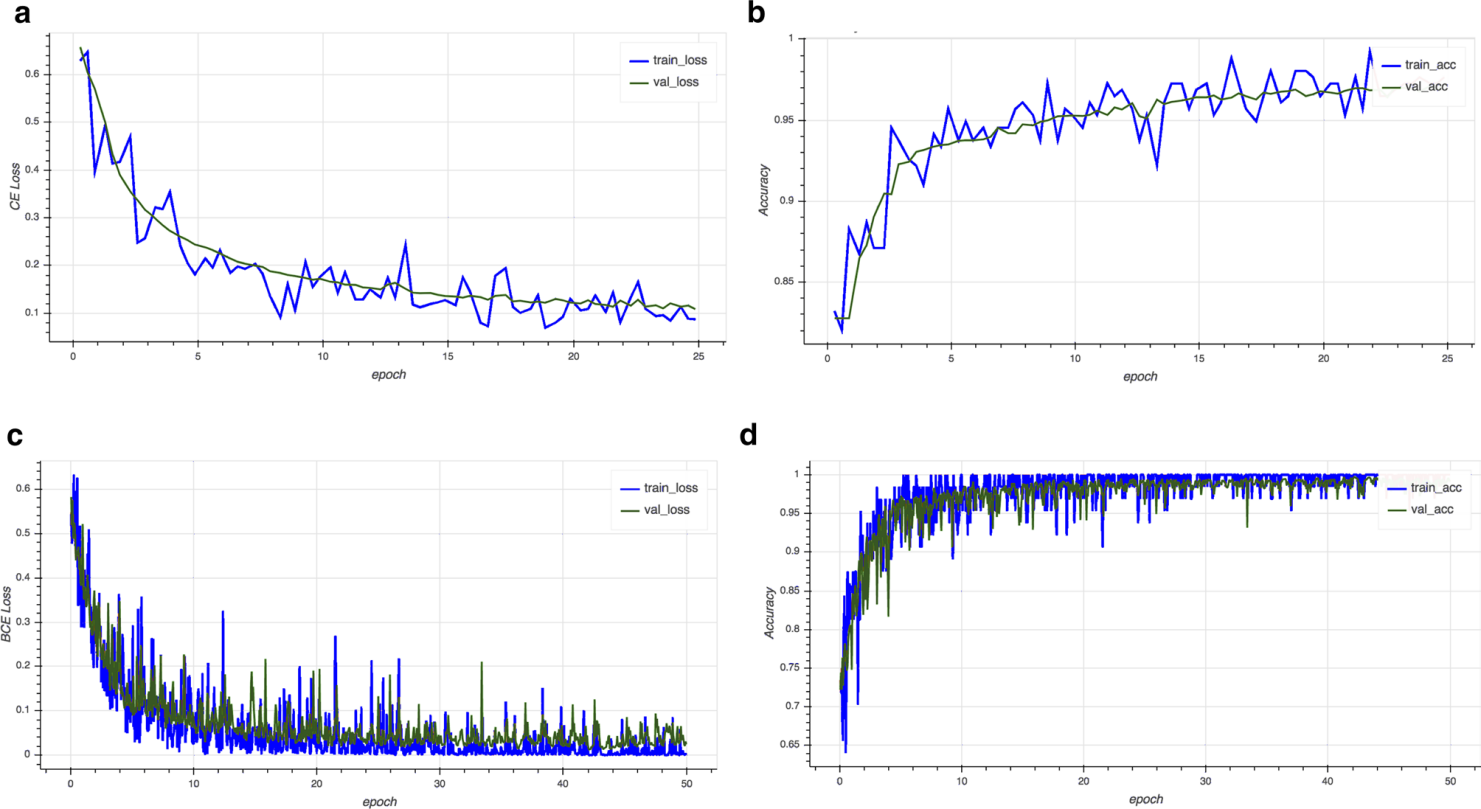
Training behavior—Convergence characteristics of the proposed DDxNet model, in terms of the cross entropy loss and accuracy scores, for the arrhythmia classification (**a**,**b**) and myocardial infarction detection (**c**,**d**) datasets.

While the proposed architecture is expected to demonstrate desirable convergence behavior even when the networks are very deep, we observed that additional regularization can be highly beneficial, particularly when training data sizes are small. DDxNet uses a weight regularization of 0.01 for all network parameters, applies gradient clipping and performs label smoothing^35^ on top of the loss function. Interestingly, we found that using dropout in the network resulted in lower performance, and hence none of the layers in DDxNet uses dropout. For binary classification problems, we used the binary cross entropy loss, and the categorical cross entropy for multi-class problems. DDxNet is implemented in PyTorch (see Code Availability) and the networks were trained using the Adam optimizer. Furthermore, we found that performing warm restarts during model training, following the idea in^36^, was beneficial in all our experiments. On the other hand, choosing different schedules for learning rate decay did not have a significant effect. All results reported in this paper were obtained by starting at the learning rate of $1e{-}4$, batch size of 64 and training for 100 epochs. Figure 4 shows the training behavior of DDxNet on two different ECG interpretation problems. As it can be observed, without any additional architectural modification, DDxNet achieves a stable convergence, even when the network is quite deep for smaller datasets.

## Data Availability

All datasets used in this were obtained from publicly released databases and pre-processed using open-source tool chains. We have added appropriate links to obtain the data as well as access the scripts for pre-processing, wherever applicable.

## Code availability

We have made the software codes for implementing DDxNet available on a public Github repository (implementation of DDxNet can be found at https://github.com/Drajan/DDxNet). Further, all pre-processing scripts were obtained from open-source tool chains, which are referenced appropriately in the “Benchmark problems and data” section.
